# Adult‐Onset BPAN: An Atypical Presentation Mimicking Early‐Onset Parkinson's Disease

**DOI:** 10.1002/mdc3.70313

**Published:** 2025-08-23

**Authors:** Mariana H.G. Monje, Padmaja Vittal, Ignacio Juan Keller Sarmiento, Lisa Kinsley, Dimitri Krainc, Niccolò E. Mencacci

**Affiliations:** ^1^ Ken and Ruth Davee Department of Neurology Northwestern University, Feinberg School of Medicine Chicago Illinois USA; ^2^ Northwestern Medicine Central Dupage Hospital Neurodegenerative Diseases Center Winfield Illinois USA; ^3^ Simpson Querrey Center for Neurogenetics, Northwestern University, Feinberg School of Medicine Chicago Illinois USA

**Keywords:** early onset Parkinson's disease, EOPD, pathogenic variant, WDR45

Early‐onset Parkinson's disease (EOPD) is defined by the onset of motor symptoms before the age of 50 and is often associated with a genetic etiology.^1^ Beta‐propeller protein‐associated neurodegeneration (BPAN) is an X‐linked form of neurodegeneration with brain iron accumulation (NBIA) caused by pathogenic variants in the *WDR45*. BPAN typically presents with neurodevelopmental abnormalities in early life with later development of severe parkinsonism, dystonia, and cognitive decline during adolescence or early adulthood.^2^ Herein, we report a unique case of a patient with a pathogenic *WDR45* variant presenting with levodopa‐responsive parkinsonism without major cognitive decline or other atypical features, mimicking EOPD.

## Case Report

The proband is a 48‐year‐old female who initially presented with 6 months of left arm loss of dexterity and progressive gait unsteadiness. She reported a history of static mild intellectual disability (ID) since childhood. She was able to graduate from high school, she held various jobs throughout her life and performed her instrumental and daily living activities without difficulties.

On examination at age 48, she exhibited asymmetric parkinsonism characterized by hypomimia, left‐sided bradykinesia and rigidity, reduced left arm swing, and narrow‐based gait with shortened stride length and pace. Her symptoms responded well to levodopa. Approximately 3 years after the diagnosis, when she was receiving carbidopa/levodopa 25/100 mg two tablets five times daily, she developed motor fluctuations and dyskinesias (see Video 1). She also had hyposmia and constipation. She had not experienced any further cognitive decline and continued to perform well in her daily activities. She had no history of developmental regression, seizures, psychiatric comorbidities, sleep disturbances or autonomic dysfunction.

**VIDEO 1:** Mild asymmetric parkinsonism with hypomimia and left‐side bradykinesia with superimposed levodopa‐induced dyskinesias characterized by involuntary choreiform movements and intermittent mild dystonic posturing of the upper limbs, neck and left foot. Gait shows mild fluctuations in step rhythm due to superimposed dyskinetic movements while walking. Marked left leg muscle atrophy is observed, attributed to a prior left ankle sprain complicated by common peroneal nerve injury and development of equinovarus with subsequent orthopedic surgery.

Her past medical history was also significant for a left ankle sprain at age 47, complicated by foot drop and progressive ankle deformity which ultimately resulted in an acquired equinovarus. EMG revealed a left common peroneal neuropathy, which was complicated by progressive left leg muscle atrophy. She subsequently underwent major orthopedic surgery to correct the deformity. Moreover, she had a history of bilateral hearing loss, beginning in her late 20s. Her family history was non‐contributory, and she is of Slovakian and Irish descent.

Genetic analysis with clinical exome sequencing identified a previously reported pathogenic heterozygous STOP‐gain variant in *WDR45* (NM_007075.4: c.400C>T; p.Arg134*). No other pathogenic variants were identified in other parkinsonism‐related genes.

**FIG 1:**
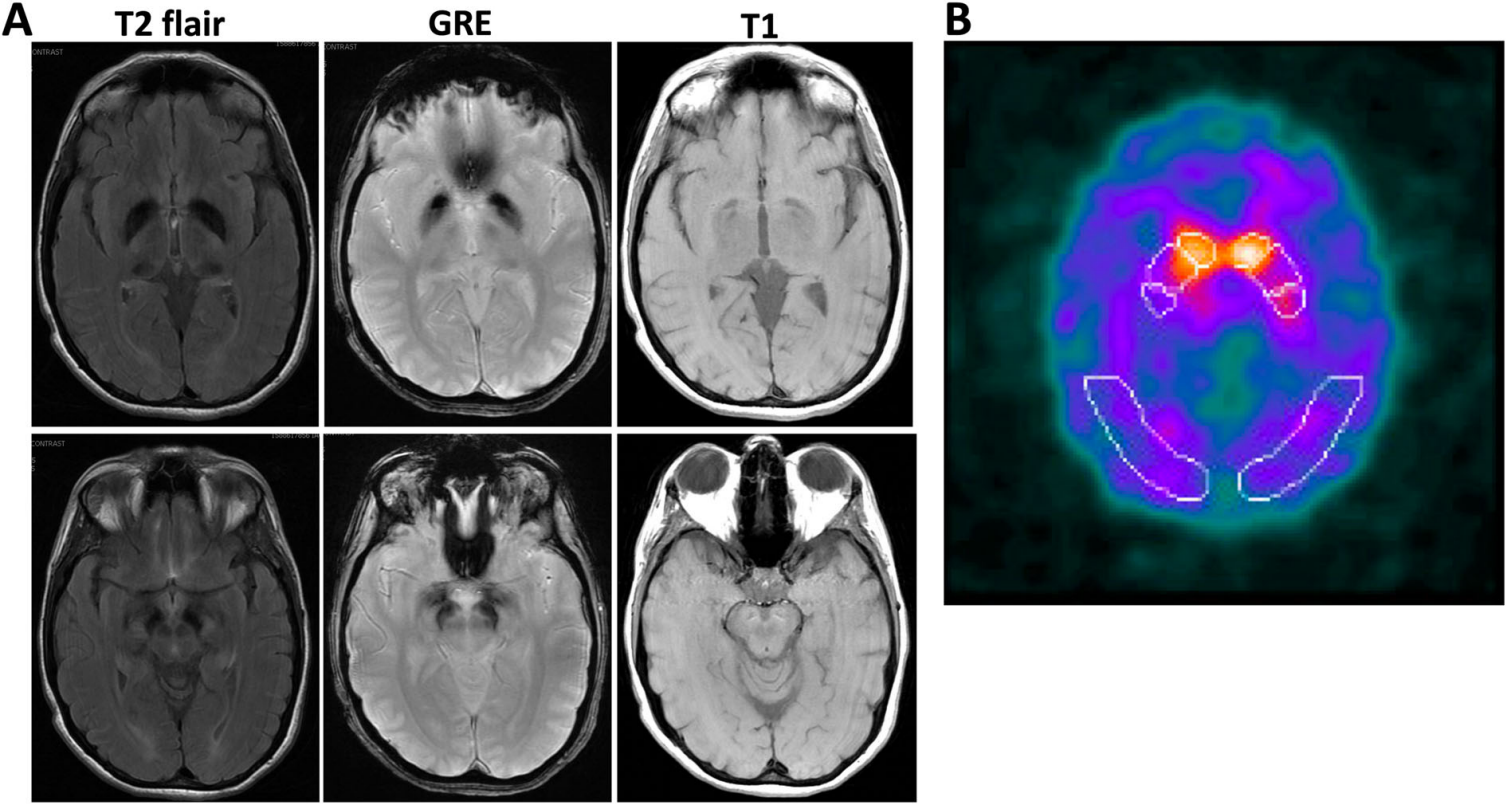
(A) 123 I‐Ioflupane single‐photon emission computed tomography (SPECT) showing bilateral decreased radiotracer uptake slightly more in the right putamen. (B) Axial T2‐weighted and multiecho gradient recalled echo (GRE) T_2_*‐weighted magnetic resonance imaging (MRI) showing bilateral hypointense signal in the substantia nigra and globus pallidus showing bilateral hypointense signal mainly in the substantia nigra but also in the globus pallidus, indicating iron deposits. Axial T1‐weighted MRI showing the substantia nigra as a thin hypointense linear region, with a partial hyperintense halo surrounding it.

Brain MRI, performed after identification of the *WDR45* variant, revealed characteristic findings of BPAN, including marked hypointensity in the globus pallidus and substantia nigra on axial T_2_‐weighted images, along with substantia nigra hyperintensity with a central band of hypointensity on T_1_‐weighted images (Fig. 1A). (123)I‐Ioflupane single‐photon emission computed tomography (SPECT) done after 1 year of symptom onset showed bilateral decreased radiotracer uptake, slightly more pronounced in the right putamen (Fig. 1B).

## Discussion

Most BPAN cases typically follow a biphasic course. In the first phase, occurring in infancy or childhood, most individuals present with global developmental delay, often with absent or minimal expressive language and significant cognitive impairment, early‐onset spastic paraplegia, and epilepsy. The severity of the developmental delay may vary with some subjects exhibiting significantly milder phenotypes.^3^ The second phase is usually characterized by progressive cognitive decline, and development of severe parkinsonism and dystonia, usually with onset during adolescence or early adulthood.^4^

Our patient, carrying a known pathogenic *WDR45* variant, deviated from this classical presentation. She presented exclusively with adult‐onset levodopa‐responsive early‐onset parkinsonism. Aside from mild static ID and hearing loss, she did not exhibit the profound developmental impairment or other early neurological symptoms typically seen in BPAN.

Late‐onset BPAN with isolated parkinsonism has been very rarely described. A subset of a few documented cases shares a similar clinical profile with mild static intellectual disability and late onset of parkinsonism resembling EOPD. These individuals are verbal and functionally independent. One case involved a 50‐year‐old woman presenting with asymmetric parkinsonism, mild weakness and spasticity, with a good response to levodopa treatment.^5^ Another report described a 31‐year‐old woman with mild static intellectual disability, EOPD and spasticity but no response to levodopa.^6^ Interestingly, in the latter case (123)I‐Ioflupane SPECT imaging showed bilateral striatal dopamine transporter uptake reduction with significant asymmetry.^6^

It has been suggested that X‐chromosome inactivation may influence the disease severity in BPAN patients.^7^ Thus, we speculate that the milder presentation of our case may be explained by an imbalanced inactivation of the chromosome carrying the *WDR45* variant. Importantly, affected females remain at risk of transmitting the complete BPAN phenotype,^8^ highlighting the need for comprehensive genetic counseling for BPAN female subjects, particularly those with a milder phenotype.

In conclusion, this case demonstrates that BPAN can present as isolated early‐onset parkinsonism, closely resembling EOPD. While WDR45 variants are very rare in cases of early‐onset parkinsonism (no additional cases observed in the PD GENEration database, which contains ~10,000 PD cases), this underscores the importance of including *WDR45* in genetic panels for EOPD screening, particularly for those cases where mild ID is present. This finding, along with similar reports of typical EOPD in carriers of pathogenic variants in other genes traditionally linked to atypical parkinsonism (eg, *PLA2G6* and *FBX07*),^9^ challenges the conventional clinical distinction between typical and atypical monogenic parkinsonism, suggesting that comprehensive genetic analysis should be considered for all individuals with early‐onset parkinsonism, even in the absence of atypical clinical features. Beyond its diagnostic implications, this observation also raises important questions about the underlying disease mechanisms. It has been shown that *WDR45* dysfunction causes cellular defects affecting pathways implicated in PD pathogenesis.^10^ Our clinical observation reinforces the concept that understanding the cellular disease mechanisms of BPAN may also be relevant to PD.

## Author Roles

(1) Research project: A. Conception, B. Organization, C. Execution; (2) Statistical Analysis: A. Design, B. Execution, C. Review and Critique; (3) Manuscript: A. Writing of the first draft, B. Review and Critique.

M.H.G.M.: 1A, 1B, 1C, 3A, 3B.

P.V.: 1C, 3B.

I.J.K.S.: 1C, 3B.

L.K.: 1C, 3B.

D.K.: 3B.

N.E.M.: 1A, 1B, 1C, 3A, 3B.

## Disclosures

**Ethical Compliance Statement:** Name of the institutional review board or ethics committee that approved the study: No IRB or ethics committee was needed for this study. Declaration of patient consent: The patient has provided written consent for video recording. This consent includes permission for the video clip and/or photograph to be submitted for publication in a peer‐reviewed medical journal. The patient acknowledged and agreed that the published material may be used by readers for educational purposes. Affirmation that all authors have read and complied with the Journal's Ethical Publication Guidelines: We confirm that we have read the Journal's position on issues involved in ethical publication and affirm that this work is consistent with those guidelines.

**Funding Sources and Conflicts of Interest:** The authors declare that there are no funding sources or conflicts of interest relevant to this work.

**Financial Disclosures for the Previous 12 Months:** MHGM is an employee from McGaw Medical Center of Northwestern University and receives support from Northwestern University Feinberg School of Medicine (Starzl Scholar Award). DK is the Founder and Scientific Advisory Board Chair of Lysosomal Therapeutics Inc. and Vanqua Bio. DK serves on the scientific advisory boards of The Silverstein Foundation, Intellia Therapeutics, AcureX and Prevail Therapeutics and is a Venture Partner at OrbiMed. NEM receives NIH funding (1K08NS131581) is supported by the Align Science Across Parkinson's (ASAP) Global Parkinson's Genetics Program (GP2). He is member of the steering committee of the PD GENEration study for which he receives an honorarium from the Parkinson's Foundation.

## Data Availability

The data that support the findings of this study are available from the corresponding author upon reasonable request.
